# Recurrent Miscarriage and Infertility Services and Supports: A Qualitative Study of Views and Experiences in the Republic of Ireland

**DOI:** 10.1111/hex.70396

**Published:** 2025-08-19

**Authors:** Laura Aoife Linehan, Marita Hennessy, Keelin O'Donoghue

**Affiliations:** ^1^ Pregnancy Loss Research Group, Department of Obstetrics & Gynaecology University College Cork Cork Ireland; ^2^ INFANT Research Centre University College Cork Cork Ireland

**Keywords:** ART, infertility, miscarriage, pregnancy loss

## Abstract

**Background:**

Recurrent miscarriage (RM) and infertility are separately associated with psychological distress and adverse pregnancy outcomes. The dual experience is devastating for women/couples but is not considered in clinical guidance for RM or fertility care.

**Objective:**

The aim of this study is to explore healthcare professional (HCP) views on the provision of care to women and couples with RM and infertility in the ROI, as well as the experiences of women who use these services.

**Setting and Participants:**

We recruited HCPs working in RM and fertility services and women with lived experience of two or more miscarriages who had undergone fertility care. Purposive sampling was used to include perspectives from multiple disciplines, lived experiences and geographical locations.

**Design:**

We conducted semi‐structured interviews, virtually and in‐person, between December 2022 and May 2023. These were audio‐recorded, and the data were transcribed for subsequent reflexive thematic analysis.

**Results:**

We interviewed 16 HCPs and 17 women with lived experience. We actively generated four central themes during data analysis. ‘Exploring all avenues’—stigmatisation and misinformation around RM and infertility impacted on seeking care. ‘Exhausting all resources’—there were physical, emotional and financial implications with this dual experience. ‘Separateness’—women felt remote from others and removed from services. ‘No woman is an island’—supports came from multiple sources in various guises but were limited.

**Conclusion:**

The experience of RM and infertility is complex, with a profound impact on the lives of women and their partners. Alongside financial and psychological supports, there is a need for greater education and awareness of miscarriage and fertility.

**Patient/Public Contribution:**

Our research group includes and collaborates with people with lived experience. Women were recruited through direct approach and social media, and their participation was voluntary with no financial remuneration. The experiences of women and their families offer keen insights into services and highlight areas requiring improvement and development.

## 1 Introduction

Recurrent miscarriage (RM), defined as two consecutive miscarriages [1], is gaining heightened attention in maternity services. The Lancet Series [2] on miscarriage has proposed worldwide reform of RM care, recognising the physical and mental consequences of miscarriage on women and their families. The authors recommended a nuanced strategy for RM care, emphasising timely access to psychological support, a departure from the former practice of reserving care until after three miscarriages [3]. This aligns with the preferences of women experiencing RM, who seek compassionate and comprehensive care [4].

The dual experience of RM and infertility and the supports required are less well‐characterised, and Clinical Practice Guidelines for RM do not consider women undergoing Artificial Reproductive Treatment (ART) [5]. Miscarriage can be a traumatic experience, which may include an established grieving period [6], and be associated with anxiety, depression and suicide [7]. Likewise, infertility is an experience known to permeate all aspects of women's lives and is psychologically, socially and financially burdensome [8]. Miscarriage and infertility are highly stigmatised conditions which perpetuate their psychological burden [9, 10]. The combined experience of RM and infertility is poorly researched, but qualitative studies are insightful. Women who experience miscarriage after ART experience negative manifestations of grief response, including depression, anxiety, frustration and anguish [11], extending to suicidality [8]. A descriptive phenomenological study of eight women described the difficulty of recommencing ART after loss and the sense of having a financial investment in each pregnancy [12]. A phenomenological study of 10 women experiencing miscarriage after ART portrayed the fear of investing in the hope of another pregnancy [13]. Women felt profoundly alone in the absence of understanding from healthcare professionals (HCPs), family, friends and others with children [12], exacerbated by a ‘social awkwardness’ around discussing miscarriage [13]. For women who experience RM and infertility and do not subsequently have a baby, the experience of involuntary childlessness may include depression and reduced life satisfaction [14]. This dual experience is deeply emotional, with widespread consequences for the women/couples affected.

The RE:CURRENT (Recurrent miscarriage: Evaluating current services) study sought to inform efforts to standardise and improve RM services in the Republic of Ireland (ROI) [15]. Alongside the review of clinical guidelines and national service evaluation of RM care [5, 16], the qualitative study found that women seek RM care within private fertility services, even without a defined fertility issue [4]. Due to the perceived restrictive definition of RM (three consecutive miscarriages), women were sometimes encouraged by public HCPs to seek investigations privately [4]. Although new legislation and clinical guidance will regulate ART provision [17, 18], currently private fertility clinics in the ROI are overseen regarding the safe care of human tissue [19], but are not mandated to report treatments undertaken or success rates [20]. The number of people undergoing ART and details of treatments and outcomes are unknown. A separate service evaluation, examining the current care practices for women with RM and infertility in clinics providing RM or fertility care across public and private sectors [21], demonstrated that RM care largely aligns with clinical guidelines, but with variation in counselling, imaging and surgical treatments offered [21]. However, poor engagement from private fertility clinics (3/9; 33%) precluded meaningful insights into fertility structures and management practices [21]. It remains unclear how many women experience miscarriage or RM within private fertility services or how RM care is delivered.

As fertility services in the ROI adapt to provide publicly‐funded ART, it is timely to examine women's experiences of RM and infertility. Few studies examine the lived experience of women/couples with RM and infertility and how management is navigated, with optimal supports unknown. The experiences of HCPs in caring for women with RM and infertility and their views on current and ideal supports and services are unexplored [22]. While aspects of fertility care in the ROI are unique, many countries do not wholly fund ART; thus, the experience of RM while undergoing private ART and the supports required may be widely applicable [23].

This study aimed to explore the lived experiences of women with RM and infertility in the ROI and to examine how HCPs providing RM and/or fertility care experience current services and supports. These insights will inform future service needs and priorities.

## 2 Methods

### 2.1 Study Design

We undertook a qualitative semi‐structured interview study using a Reflexive Thematic Analysis approach [24, 25] (Supplementary File 1). We embedded a constructivist epistemological approach, recognising the subjective nature of an individual's experience of a phenomenon, alongside an inductive (data‐driven) analytic method [25]. Our study and positionality were informed by feminist principles, appreciating the social and cultural context and the centrality of power, on women's lived experiences and health service provision [26].

### 2.2 Ethical Approval

Ethical approval was granted by the Clinical Research Ethics Committee of the Cork Teaching Hospitals [Reference number: ECM 4 (J) 28/06/2022 and ECM 3 (eee) 13/12/2022].

### 2.3 Recruitment

We purposively sampled individuals involved in RM and fertility services and women with lived experience of RM and infertility. We sought HCPs in both private and public sectors likely to encounter these women, such as consultant obstetricians, fertility specialists and clinical midwife and nurse specialists. HCPs were recruited through the research team's national networks and contacted by phone or email. To enhance recruitment, we used snowballing, where participants identified other potential participants [27]. In this instance, the first participant sought permission from the potential participant before sharing contact information with the researcher.

We included women with lived experience of two or more consecutive miscarriages, in line with women's preferences and European guidance, but limited to consecutive to align with the Irish context [4, 28]. We included women whose most recent miscarriage occurred 6–48 months before participation, to allow sufficient time from the loss and allow for potentially longer times between pregnancies for those undergoing treatments. We included women who experienced infertility before RM and women who experienced RM and subsequently had secondary infertility or sought fertility care. Women were recruited directly via HCPs nationally, or social media, including through the Miscarriage Association of Ireland site. We used the concept of ‘information power’ to determine when to cease sampling, stopping when the major categories showed depth and variation [29].

Participants were provided with an information leaflet, consent form and research team contact details. Participants who were approached directly were sent two reminder emails. Details of participation in the study were outlined to potential participants upon contact with researchers. Written consent was obtained from participants before their interview, either in‐person or electronically, according to interview type. Participants were reminded that the interviews were voluntary and that withdrawal was possible for meaningful and informed participation [30]. Participants were reassured that they could pause the interview, given the subject matter.

### 2.4 Setting

We sought perspectives from different locations across the ROI; therefore, our recruitment efforts were inclusive of different settings (hospitals/clinics), hospital groups, types/sizes of maternity units or hospitals (tertiary/regional/peripheral), types of RM services (dedicated RM/pregnancy loss clinics/non‐dedicated services within gynaecology services) and types of fertility services (public/private) (Supplementary File 2).

### 2.5 Data Collection

Participant views were explored using semi‐structured interviews, conducted via Microsoft Teams or in‐person between December 2022 and May 2023. Microsoft Teams, widely used since Covid‐19, enhanced accessibility [31]. Topic guides, informed by previous research and clinical experience, were piloted and revised within professional networks [4, 22]. Two guides were used; one for women with lived experience and another for HCPs (Supplementary File 1). The interviews were conducted by L.L., with M.H. attending the first interview for each type of participant. One interview was conducted by M.H. as the participant was known to L.L. Interviews were audio‐recorded. Field notes and regular debriefings were undertaken and used to inform the analysis and assess information power.

### 2.6 Data Analysis

The audio files were transcribed verbatim by a professional transcriber. Confidentiality and data processing agreements were in place, as was a distress protocol. Transcripts were verified against the recording, pseudo‐anonymised, coded and imported into NVivo 12 for data management [32]. Data was analysed using Reflexive Thematic Analysis [24]. Patterns (i.e., themes) were identified through a rigorous process of data familiarisation, data coding, theme development and revision (Supplementary File 3) [24, 25, 33]. We analysed data from women with lived experience and HCPs collectively to capture diverse perspectives.

### 2.7 Ethical Considerations

The research team is experienced in working with ‘vulnerable’ populations and sensitive topics, including pregnancy loss. L.L. is a clinician experienced in working in RM clinics [4, 34, 35, 36, 37, 38]. In addition to ongoing informed consent, a distress protocol was prepared. This encompassed an end of interview debrief to ensure participants' well‐being and contacting a family member/friend for support if distressed, with further follow‐up within 24 h. Support network details were provided in the information leaflet and reiterated during the interview. If further support was required, the bereavement midwife in the relevant maternity unit was contacted, with the participant's permission.

### 2.8 Reflexivity Statement

The research team included an obstetrician and PhD student (L.L.), a public health/health‐services researcher (M.H.) and an obstetrician/maternal–fetal medicine subspecialist (K.O'.D.), who discussed their positionality and engaged reflexively throughout the study. L.L. and K.O'.D. are involved in the care of women/couples with pregnancy loss. All three researchers authored the Irish National Clinical Guideline for RM. K.O'.D. and M.H. are experienced in qualitative research; L.L. received training and mentoring in qualitative methods and interview techniques.

## 3 Results

### 3.1 Participant Characteristics

Between December 2022 and May 2023, we conducted 33 interviews with 16 HCPs and 17 women with lived experience (Table 1). No participants withdrew from the study. Four interviews were held in‐person and 29 virtually. Among the eight consultants interviewed, seven had subspecialist training in fertility and one focused on pregnancy loss. The fertility consultants had current or previous experience in private or not‐for‐profit fertility clinics. One Clinical Nurse Specialist in fertility was trained as an Advanced Nurse Practitioner.

**Table 1:** Details of HCPs and geographical locations.

HCPs		(*n* = 16)
Consultant obstetrics and gynaecology		**7**
Trained in fertility		6
Working in pregnancy loss		1
Fertility specialists (private)		**1**
Fertility clinical nurse specialist		**4**
Public		3
Private		1
Clinical midwifery specialists in bereavement and loss		**4**
Geographical location (*n* = no. of hospitals in region)	HCPS (*n* = 16)	Women with RM (*n* = 17)
Ireland East/South East (*n* = 3)	**5**	**9**
Ireland South/South West (*n* = 4)	**7**	**5**
Ireland West (*n* = 3)	**3**	**2**
Ireland North/North West (*n* = 1)	**1**	**1**

*Note:* Bold values indicate the total no in each sub‐group

The women interviewed were aged 35–47 with a median age of 39; all were white Irish, with third‐level education and in professional employment. Women had experienced between two and nine first‐trimester miscarriages; third and subsequent miscarriages were not necessarily consecutive. Two women had an additional second‐trimester loss after RM. Ten women had at least one living child, and two women were currently pregnant. All but one woman, undergoing ART with donor sperm, were in heterosexual relationships. Five of the seven women with no living child were still seeking fertility care. Interviews lasted 29–57 min (mean 42) for HCPs, and 33–80 min (mean 61) for women.

We generated several themes and sub‐themes through the analytical process (Table 2). Supporting quotes are provided within Tables 3, 4, 5, 6, where P = Person with lived experience; SP = Service Provider; W = Woman; M = Man.

**Table 2:** Overview of themes.

Themes	Sub‐Themes
Exploring all avenues	Knowledge and awareness
	Women wanted to understand why
	Navigating the limited evidence
	Treatment was a complex undertaking
Exhausting all resources	Burden of RM and infertility
	Psychological impact
	Giving it their all
Separateness	Remote within the system
	Remote from others
	Remote partners
No woman is an island	Supports were limited
	Support came in many forms
	Staff wanted to do more for women

**Table 3:** Theme 1 ‘Exploring All Avenues’.

Sub‐Theme	Illustrative quote
1.1 Knowledge and awareness	I just thought it was a guaranteed process as well you know like you only, just deciding to do IVF that is the hardest part, the rest is easy peasy. [PW17]
	It happens to people you know the more people you talk to, but it is not really talked about, nobody talks about their experiences of it, even me, even me now like you know I still don't, and I am probably hypocritical in that way. [PW15]
	…if you want to have three children without resorting to IVF treatment… you probably need to start or have your first child at age 23, so it is an awful lot younger than even we would realise. [SPW11]
	…where the woman is over 40, I do discuss donor eggs. It is always an uncomfortable conversation and I say that I am not saying that this is the absolute next step, but I say it just in case no one else says it to you and you are 45 and you are in this position. [SPW4]
1.2 Women wanted to understand why	What, what is the reason behind it? Is there something wrong with me or is there something wrong with my husband? I don't know anything medical; I am not from a medical background. I never knew anybody to suffer two losses. [PW14]
	I just felt the whole experience was being minimised and not really, the impact that it was having on me wasn't being heard because somebody decided somewhere that you need three. [PW15]
	It is this perception that the hospitals or ‘stick in the mud’ doctors aren't really doing enough for them so obviously that is untrue you are trying to keep them safe and not expose them to exploitative tests and therapies, but people do like tests. [SPW6]
	I read an awful lot, like I am pretty sure it came to a point at which I could have taken exams in reproductive immunology and probably passed them. [PW11]
	I don't know if I was to have another baby and I didn't take the medication that I took this time would I have another loss or would the outcome be different. It is just one of those things I am going to have to live with, I am never going to know, and I wracked my brain trying to figure out what it was but sure I couldn't. [PW14]
1.3 Navigating the limited evidence	I understood that this is really cutting edge stuff like and while I definitely understood what they were saying to me here in that we don't want to take on treatments that are so new and so experimental and don't have an evidence base behind them, I get that, but as a patient in a situation where you are literally hanging on to desperation you want those experimental treatments, you want the tests done. [PW11]
	I don't want someone to put a strait jacket on me and say, ‘we are coming up with a protocol and you can only practice this way and no other way’. I think that is the death knell of medicine and I think that the new generation coming up have the blinkers on of evidence based medicine, follow the evidence, if there is no evidence you can't even do it, no, no you declare the evidence is there but we are trying something new and patients love to try something new if nobody has the solution to their problem. [SPM1]
	…you know just to have a national pathway and I suppose with this hub that will help to have somewhere whether they are all treated the same really. That everyone gets the chance of the investigations because you know like you say it depends on where they are and what area they are in and have they access to private care or not. [SPW12]
1.4 Treatment was a complex undertaking	…what do you do like, do you kind of find out that maybe you mightn't have to go down the road of IVF but you might have to just see maybe is there anything further wrong or … see maybe if there is something that I didn't know about. It is a step into the unknown I suppose but where else are you to go… [PW12]
	To be honest I never struggled with the medical side of it, the only thing I really struggled with was the waiting and the not knowing … in all of the journey that was worse than any physical impact. [PW17]
	For some couples they will come and explore. They will do the counselling and when it comes to actually executing and moving forward, they will sometimes find that it is actually just too much for them and they will withdraw and they will go the adoption route instead or do nothing, which is equally very sad. [SPW9]
	I just couldn't imagine not being a mum. [PW1]
	I would write it (the options) down on a piece of paper for them and then really the last option is to gently introduce, just to live and love each other and look at other aspects of life … to put this chapter behind them. [SPM3]

**Table 4:** Theme 2 ‘Exhausting All Resources’.

Sub‐Theme	Illustrative quote
2.1 Burden of recurrent miscarriage and infertility	I remember one day I was like at my computer, and I just started crying … after doing two rounds of IVF back to back, I would never do that again because I was in an awful state. I was so busy in work, I was taking half days here and there … because … I had to go to Dublin … because they needed blood test the same day … to know whether to alter my medication. So, I had to drive … 5 h before then I would go and work for the day … it was just so busy in work as well and everything and things going wrong. [PW2]
	I felt like I was … a drain, do you know? I felt like … I had this invisible thing. [PW1]
	…what bothered me was every time you went down to get anything done, there wasn't a conversation about how much this would cost but there was an invoice afterwards and again … it could be two hundred quid, you know your DNA fragmentation is two hundred and eighty… one of them was like an embryo glue that they used and that was five hundred quid … it was all of these five hundreds you know. [PW17]
	they would be the most amazing parents and give children the most beautiful home and it is just not an option because they don't have the cash. [PW7]
2.2 Psychological impact	I was three and a half years you know trying and I suppose two and a half years having treatment constantly and never having a pregnancy and I wouldn't consider, I would consider that like an even darker place to be than miscarriage and recurrent miscarriage. I think, I suppose, by the time I had miscarriages and stuff I was already down it wasn't like ‘oh my god something bad has happened to me I can't believe it’ like I could believe it very well. [PW9]
	So I did go into it with quite a bit of hope … but hope and IVF are a kind of dangerous thing … you do have to have some positive attitude … and yet it is a knife edge thing, getting your hopes up too high is very dangerous … because the let down when it doesn't work is just enormous. [PW13]
	It just takes time to get over and months and months even down the line … it still affects you and everybody is so different and the three I had were so different and how I dealt with them … I don't know hormones as well are probably just all over the place and the loss obviously erm just the disappointment, just every emotion, just sadness, just pure sadness and very lonely thing to go through I just remember feeling so lonely even though I had people around me just yeah, nobody really understands unless you are going through it you know. [PW15]
	when I bought the stuff, I asked in in the shop you know if we lose the baby, you know can we give them back, because I always just felt like you know, that could happen. [PW5]
2.3 Giving it their all	I just feel that before I give up, I have to throw everything at it. [PW09]
	…the sense that I always got was that it was not well understood and even with the treatment the aspirin, the blood thinners and the steroids like that is just ‘sure we will try this, we don't really know if it works or not’. [PW5]
	I have it etched into my head, even my numbers from my HCG even two and a half years ago, I remember everything like from my head because I was just like devoted to it for so long … everything I know off hand, dates, like measurements of my blood, medication that I have taken, that I have taken before that I have stopped taking, like milligrams of medication. [PW14]
	At that point I just knew that I couldn't physically or mentally put myself through anymore at that stage, up until then like over those ten or eleven years we had kind of just gone from one cycle to another to another, straight after each other or as close as possible that we could, and I just couldn't. [PW11]

**Table 5:** Theme 3 ‘Separateness’.

Sub‐Theme	Illustrative quote
3.1 Remote within system	I find that there is a massive conflict between the private/public issue and I suppose it is conflict and it is misinformation and it is also the different health care providers input so I think it is a very mish mash set up. [SPW4]
	Ahh it is very disjointed, and actually even when we look at it from the private point of view, if you have your fertility treatment privately and you miscarry, you are coming into a public hospital, (people) you don't know, who haven't met you so yes, it is very, very disjointed, it is a postcode lottery definitely. [SPW8]
	There some issues in terms of bleeding and I was supposed to be taking the medication. I was getting on to them in Spain and I was asking them like should I keep taking the medication if there was spotting and stuff like that and she said no, not to do it, and then I went in for my six‐week scan into the hospital and they said no, you must take your medication, so then I was all confused. I was ringing my GP then and anyway between the jigs and reels then I suppose I was referred to a consultant. [PW12]
3.2 Remote from others	I felt like no one knew what to do with me, I felt I was no longer following advice, I felt like I was just … doing my own, doing my own thing. [PW1]
	I was pregnant at the time and I met one of my friends, and then, when I told her I had miscarried again, she just bawled crying like. I just said, I can't be dealing with this, other people being so upset let alone myself. [PW2]
	… for the last few years it has been people having babies, now it is starting to turn into they are celebrating various milestones as they grow up and it is first day at school and communion days and whatever else. As all of my contemporaries grow and their children grow, it is just each different year there is something else I am not getting or missing out on. [PW11]
3.3 Remote from partner	… we were always kind of on the same page in terms of keep trying but he always said you know if I wanted to stop that he would be upset but like it was kind of up to me because you know I am the one doing all the heavy lifting. [PW5]
	My husband erm doesn't speak, I would say he is even more depressed than me, erm he finds it so difficult, and he doesn't verbalise it. He never speaks to anybody about it, he has isolated himself from his friends really and… I think he finds the kind of explosion of babies that happens around you know our age really, really difficult. [PW9]
	… a lot of times I will have the man in the couple almost despondent with me, can't make eye contact, just they have reached the bottom because we are now talking about this donor that is going to, in their eyes, replace them you know. [SPW9]

**Table 6:** Theme 4 ‘No Woman is an Island’.

Sub‐Theme	Illustrative quote
4.1 Supports were limited	With AHR bill coming through at least in draft form there is quite an emphasis on counselling, and you start to wonder who is going to be paying for this, and how that is going to be funded, and there are a limited amount of counsellors who are mostly privately run in the country. [SPM2]
	I will never forget it we were leaving the hospital and there were these women, and they were going home with their babies and you were going home with nothing. Ahh it was just … it is like an out of body experience, it is just, oh it is horrible, like. [PW8]
	To be honest when you know I was in such a low place I didn't have the mental…. I don't know I wasn't even capable of making a phone call and making an appointment you know I was just so incredibly fragile. So even if something like … basic stuff, some businesses will follow up a week after you know ‘are you happy with your car service?’ … I mean a phone call a week later to say ‘how are you doing, are you okay you know would you like an appointment with…?’ I mean something like an automatic appointment with the bereavement, with the counsellor or something that you could always turn it down if you didn't want it. [PW4]
	I know it is common and I know it happens but equally to that parent that is their baby and you wouldn't deal with somebody who was after losing a family member like ‘ah well DNA’ you know it would be ‘okay’, even just to sit down and offer them a listening ear for 10 min that is all you want you know to kind of think that you are not just next, next, hurry up. [PW16]
4.2 Support came in many forms	…a bereavement midwife was the first person I met and she gave me her details so that I could contact her if I needed anything and I saw the registrar followed by the consultant. That was super constructive, it was really helpful, and I felt listened to, supported, I felt like there was a plan going forward, that was a fantastic service, absolutely brilliant. [PW10]
	I like that podcast listening to other people's stories and you have to be in the headspace for it like there were certain times I wouldn't be able to listen to it, but they always say the supports afterwards and all that, that has been good as well just to know you are not the only one, that is a big thing for me like. I felt I was the only one who had gone through this there are hundreds you know thousands of women out there. [PW8]
	The last time I just rang my boss, and I … told her what happened she was really, really supportive, really good and you know that was a big relief, it really helped with being able to kind of concentrate on getting back to normal I suppose again. [PW15]
	By pure fluke on Instagram, I came across the (support group) who just happened to be having a meeting that night … it was hands down the best thing that I have done, hands down, like just to speak to like‐minded people was just incredible. [PW17]
4.3 Staff wanted to do more for women	We have to see the wood from the trees and protect them from things that we know might do harm, may, may, have benefit but the evidence isn't there to robustly support it and also costs a lot of money. [SPW1]
	…my grave concern about that is how well staffed they are going to be because if you don't have quality staff, the quality of what you are going to produce is going to be compromised. [SPW11]
	I did speak to my director … I would love to facilitate an early pregnancy loss support group because they often say ‘God you know you just feel so on your own’ and I feel it would be great for women in the same area just to have something. [SPW13]

#### 3.1.1 Theme 1. Exploring All Avenues

Women were blindsided by the experience of RM and infertility and did not anticipate it could happen to them. They searched for answers as to why this occurred and what they could do to try and improve their chances of having a baby (Table 3).

##### 3.1.1.1 Sub‐Theme 1.1 Knowledge and Awareness

Mostly, women did not envisage RM or infertility. Expectations were based on the seemingly ‘easy’ experiences of peers or their own previous successful pregnancy. Some women had awareness of miscarriage and accepted one miscarriage as being ‘the one in four’. For all women, a second miscarriage was unexpected, indicating that something was wrong and ignited fear around their reproductive health. For women who had undergone ART to conceive, miscarriages were devastating, as women did not foresee issues after conception. Women with RM who chose to undergo ART did not expect that ART might be unsuccessful, anticipating issues with a pregnancy rather than conception.

As women processed the shock of miscarriage, they realised their unawareness of miscarriage and wider fertility issues. In acknowledging this naivety, women questioned the lack of societal attentiveness to miscarriage and infertility and their increasing risks with advancing age. Women were perplexed by the stigma that still surrounds miscarriage and appreciated that some of their behaviours were due to stigma.

Participants (to mean both women and HCPs) recognised that awareness facilitates informed decision‐making and that reproductive health education should begin in school. Women expressed regret for delaying starting a family, with a sense of running out of time. HCPs identified unawareness of the links between maternal age, fertility and miscarriage and that many women were not only uninformed but also misinformed by online resources. HCPs highlighted that the low success rates of IVF, especially for older women, are not widely known, and how celebrities becoming pregnant in their late 40s set unrealistic expectations.

Women expressed that information provision was often poor or delivered late in their journey. All participants identified that HCPs had variable knowledge of miscarriage care, RM or infertility. HCPs identified fertility as a significant omission in midwifery education. This inexperience impacted women's care throughout their journeys. Inadequate explanation of miscarriage and miscarriage management, with little or no written information or resources, added to the trauma of miscarriage and was perceived as apathy by women. Fertility treatment often lacked adequate explanation or signposting, prompting self‐directed research. Women valued honest, clear information and direction when provided. HCPs with awareness felt obligated to comprehensively counsel women to ‘protect them’ from misinformation going forward.

##### 3.1.1.2 Sub‐Theme 1.2 Women Wanted to Understand Why

Women wanted to understand why they experienced miscarriage or infertility. The first natural step for many women was to seek answers from HCPs. Such reasons for miscarriage or infertility could then hopefully be ‘fixed’. Moreover, women perceived that a reason for the loss would ease some of the uncertainty faced and was linked to women's fears of not having a baby.

However, investigations were not easily obtained and often did not provide the desired answers. Investigations were perceived to be withheld when performed after three miscarriages only, with disregard for ‘just two’ pregnancy losses. Even after investigation, due to the limitations of evidence‐based investigations for RM, many women felt under‐investigated or not reassured by ‘normal’ results. Conversely, HCPs felt a desire to ‘protect’ women from unnecessary or unproven investigations but acknowledged the difficulty in accepting a diagnosis of ‘unexplained’ RM or infertility.

Where investigations were not performed or provided insufficient answers, women felt a need to ‘explore all avenues’ for information. They progressed from their general practitioner (GP) to the RM clinic or public fertility services, then advanced onto private fertility services, potentially multiple private providers. This sometimes included travelling abroad to obtain investigations or expertise. This care‐seeking was heavily supplemented with online research.

Women found the concepts of ‘chance’ or ‘bad luck’ to be inadequate explanations, instead choosing to ascribe miscarriage or failed cycles to aberrant results or their own bodies. Where women accepted the lack of answers or received a reason (e.g., aneuploidy), a frustration remained over their inability to control outcomes.

##### 3.1.1.3 Sub‐Theme 1.3 Navigating the Limited Evidence

Women found that, alongside definitions, other aspects of RM/fertility care were constrained. Women were frustrated by the limited evidence‐based investigations and treatments. While some accepted their clinician's counsel and chose to remain within ‘safe’ boundaries, other women who had exhausted available evidence‐based treatments sought to circumvent these boundaries. Women understood treatment‐related risks but felt that their exceptional circumstances merited exceptional treatments. This sometimes resulted in confrontation between clinicians and women/couples regarding care. Conversely, some women described discomfort in clinics abroad, which they found ‘so ridiculously unregulated’.

The absence of clinical guidelines for RM or fertility, or fertility regulations, resulted in variation in care received nationally. HCPs recognised this contradictory situation, whereby women were denied basic guidance‐based investigations in some centres and freely offered unrecommended treatments in others. HCPs knew that ‘sticking to the evidence’ and refusing to use unproven tests or treatments meant that women might pursue care elsewhere, despite the potential risks. The need for standardisation and equal access to expertise in this challenging clinical scenario was identified by women and HCPs.

##### 3.1.1.4 Sub‐Theme 1.4 Treatment Was a Complex Undertaking

Women recognised that fertility treatment was a significant undertaking, and it was not pursued lightly. After miscarriage or infertility, however, it represented a positive, active step. Women understood that, within treatment, difficult choices may await and tried to protect themselves by setting their own boundaries or expectations.

Medication regimes were complex and an encumbrance, physically and emotionally, due to significant side effects. Even where women managed the regimes well, the uncertainty of treatment was an emotional toll, which women admitted to underestimating. Waiting to discover if treatments worked was taxing, and for women with RM, the first trimester was fraught with anxiety.

After unsuccessful treatment, women/couples faced challenging decisions. Gamete donation was an impasse, particularly without anonymous gamete donation in the ROI. Women reported that male partners especially struggled with sperm donation. Most women/couples chose to exhaust all other options before considering gamete donation. Couples explored adoption but deemed it unviable as the process was complicated and very limited.

The decision to cease treatment was usually protracted. Generally, women/couples had explored all treatment options, depleted emotional or physical resources or reached a pre‐set boundary (e.g., donor gametes). Women struggled to stop trying if treatment options remained. The decision appeared to be better accepted if the woman/couple determined treatment cessation. While some fertility clinics set boundaries on treatment options, most HCPs felt their role was to inform and not to impose.

#### 3.1.2 Theme 2. Exhausting All Resources

Women channelled substantial energy and resources into having a family—‘gave it their all’. While RM and infertility were private burdens, the experiences infiltrated their lives, including their work, finances and mental health (Table 4).

##### 3.1.2.1 Sub‐Theme 2.1 Burden of RM and Infertility

RM and infertility were personal experiences that women/couples did not share beyond close friends and family, or sometimes, at all. Most women attempted to continue working throughout the experience, partially due to the need to maintain normality and privacy, but also due to the absence of paid reproductive leave. Women struggled to continue their lives as normal during this time. They sometimes lacked concentration at work due to emotional stressors, such as grief after miscarriage, anxiety about a current pregnancy or ART, or medication side effects. Occasionally, women returned to work immediately after miscarriage due to concerns over accrued sick leave. Juggling fertility appointments was another source of stress, with appointments squeezed into early mornings or annual leave. Women living in rural or western Ireland often had significant travel burdens. Women felt uncomfortable disclosing their reproductive history at work, but were equally uneasy about lying regarding sick leave. When women did disclose their experience, they were generally supported. Women employed by companies that provided reproductive health leave found it immensely helpful.

Life outside of work was similarly affected. Women struggled to be onlookers to the happy events of family/friends while experiencing RM and infertility, and many withdrew socially. Moreover, they felt their own lives were suspended. They felt paralysed and unable to make decisions regarding work, housing or even holidays abroad, as these could be impacted by treatments or pregnancy.

Women contended with the unexpected undertakings associated with RM and infertility. This included the time taken to organise appointments, travel, do additional investigations and chase results. While women understood how expensive treatment was, they did not anticipate the cost of add‐on treatments, multiple cycles and hidden expenses such as medication, travel, childcare and unpaid leave. Women were acutely aware of these costs, recognising the privilege in managing to afford treatment and the injustice of a ‘two‐tier’ (i.e., public‐private) system.

##### 3.1.2.2 Sub‐Theme 2.2 Psychological Impact

Women struggled with the grief of repeated miscarriages and the unmet desire to have a family. They underwent a rollercoaster of emotions associated with undergoing treatments and medication side effects. As per sub‐theme 1.1, women were bewildered by RM after treatment. Trying again after RM and infertility was not undertaken lightly, as women knew how stressful treatment was, and how devastating failed cycles and miscarriage were. They hoped fervently that they would have a baby, and this helped them to re‐embark on their journey. However, hope was counter‐balanced by the fear of never having their desired baby, which was another powerful driver for trying again.

Consequently, women experienced mental health issues, particularly anxiety and depression. Women were especially anxious in the first trimester of subsequent pregnancies and when undergoing ultrasounds, but many found their overall anxiety was heightened. Women remained anxious throughout any pregnancy after miscarriage and were fearful of being excited, anticipating the worst. This was recognised by HCPs and the need for support and engagement with perinatal mental health. Sometimes, it was only after pregnancy or treatment cessation that women experienced a deterioration in mental health or felt that they had the capacity to seek professional help.

##### 3.1.2.3 Sub‐Theme 2.3 Giving It Their All

Women wanted to ensure that they had tried everything to have a baby. After ‘exploring all avenues’, women sought out providers who would offer their researched therapies. Women sometimes tried medications and treatments of uncertain efficacy or safety, justified by a need to try new things and to ‘throw the kitchen sink’ at treatments. Similarly, women trialled alternative therapies and diets that they were sceptical of.

Women poured all their energy into efforts to have a family. Alongside the normal demands of life, they followed treatment regimens carefully and attended for investigations on schedule. To varying degrees, women became fixated on the measurements and monitoring involved, which were stressful. This stress was augmented by delays in completing investigations, which incited women to self‐advocate between providers. Women pushed boundaries on investigations and treatments, set by themselves and clinicians, as their journey progressed. Overall, the journey was incredibly demanding, and women found that they were pushed to their physical and mental limits, which sometimes resulted in treatment withdrawal.

#### 3.1.3 Theme 3. Separateness

Women identified that their dual experience set them apart in the healthcare system, caught between public RM care and private fertility care. They felt that it placed them at a remove from family and friends, and sometimes, their partners (Table 5).

##### 3.1.3.1 Sub‐Theme 3.1 Remote Within System

Participants identified that women with RM and infertility were caught between the public and private systems, with the divide complicating their experiences. This occurred regardless of whether women had public RM care first and then sought private fertility care, or vice versa. This was burdensome as women had to navigate appointments between both systems and ensure their records were transferred. This was augmented for women seeking care abroad or by GPs declining to perform blood investigations requested by private clinics that were ‘outside of their remit’. Women attending public hospitals for miscarriage or gynaecological care after ART felt like outsiders, partly for ‘cherry‐picking’ the RM investigations they attended for, and partly because their ART was only available privately. Women found attending maternity services for RM care upsetting, as encountering babies and pregnant women felt unavoidable.

HCPs recognised that women/couples with RM and infertility did not ‘fit’ the system, their specific needs were unmet and provided guidance in navigating the systems. HCPs identified that care was ‘disjointed’; this referred to a lack of standardisation of care, the urban centralisation of care and a lack of networking/communication framework between the systems. There was a similar disconnect with urology services regarding male factor infertility. HCPs acceded that while women with RM should not have to attend private fertility services, considering the limited public services and the age profile of women affected, this progression was not unreasonable.

##### 3.1.3.2 Sub‐Theme 3.2 Remote From Others

Women with RM and infertility felt remote from other women attending RM/fertility services, recognising that they were outliers and that their experience was atypical. This realisation that they were outside the expertise of, or services available to, clinicians propelled ‘researching’ and subsequently, self‐advocacy as they gained knowledge.

Women were reluctant to share the burden of their experiences and felt isolated. Due to the emotional and medical complexity, they believed that family and friends ‘wouldn't get it’. Sharing the experience further burdened women with well‐meant enquiries and emotional reactions. Although women chose to keep the experience private, they still keenly felt the isolation and loneliness.

Women and their partners felt separate from fertile couples and could not help but compare their experiences. They envied their seemingly easy conceptions and pregnancies and the nonchalance with which they treated pregnancy. Again, they felt ‘in limbo’ and found it difficult to be onlookers, while others progressed and achieved all that they had hoped for. Being ‘left behind’ was more than a feeling; many women found that relationships drifted due to the differences in circumstance.

##### 3.1.3.3 Sub‐Theme 3.3 Remote Partners

Male partners were the main support for women during RM and infertility, and while it was a shared experience, there were notable differences. Participants spoke about how partners did not have the same physical experience of either RM or ART. Partners did not press women to persist and were often more willing to cease treatment. Women sensed that although their partners felt each loss, they did not grieve equally.

Men were treated differently on the journey. Women and HCPs identified them as ‘onlookers’ to miscarriage and ART, but conceded that this could be as traumatic. Participants perceived that men were less inclined to talk about their experiences. They acknowledged that medical appointments and supports were largely woman‐focused. Male infertility was perceived to be a painful issue; participants reported that men felt guilty for the couple requiring ART. Sperm donation was an especially emotive dilemma.

Couples recognised the impact of their ordeal and turned to each other. Women worried about their partners and supported them. Likewise, the male partner was acknowledged as a stalwart support and a safeguard in questioning their journey and the treatments undertaken.

#### 3.1.4 Theme 4. No Woman Is an Island

This theme encompassed the contrasting experiences of people; women took comfort in and embraced various supports from different sources but also felt a need to retreat from unsupportive environments and behaviours (Table 6).

##### 3.1.4.1 Sub‐theme 4.1 Supports Were Limited

Women with infertility had few psychological supports provided publicly, with the fertility nurse specialist providing most support. HCPs were acutely aware of this need for specific fertility‐related psychological supports, including psycho‐sexual counselling. Women in the private system did have access to counselling supports, but many found they could not undertake this while focused on treatment. Clinic policies varied from mandatory to no counselling.

As evident across other themes, a lack of knowledge and awareness among HCPs regarding RM and infertility heightened women's concerns and led to missed opportunities for supportive care. Women were typically not offered miscarriage support until they had experienced RM, usually three miscarriages. Clinical care for individual miscarriages could be poor, which compounded their distress and sense of being unsupported. Women identified ultrasounds as very stressful. This was exacerbated by repeated scans for uncertain viability or insensitive provision of a miscarriage diagnosis. Women pinpointed miscarriage management as problematic. Discussions with HCPs regarding their options were often rote and indifferent. Women felt medical management was inadequately explained, with the anticipated pain and bleeding understated, ‘like a heavy period’, resulting in traumatic and frightening experiences. Women desired a clear explanation of their care plan with written information. Women found the lack of separate spaces at all stages of miscarriage care—diagnosis, in‐patient stay or emergency care—distressing. Critically, for women attending private services, the lack of out‐of‐hours private care meant their first interaction with public services was while experiencing a miscarriage or medical emergency. The unknown setting and absence of a familiar caregiver added to their unease.

Occasionally, women had to fight HCPs to receive their recommended supports, like serial hormone levels or early scans. Some hospitals provided no follow‐up, even after a second‐trimester loss, as there was no pregnancy loss service. Women sometimes pursued support or follow‐up appointments themselves. In fertility services, women reported feeling ‘on a conveyor belt’ and that miscarriages were to be managed swiftly to recommence ART. Participants reiterated the lack of support for men at all stages.

##### 3.1.4.2 Sub‐Theme 4.2 Support Came in Many Forms

Women did have positive experiences of care, and many forms of support were helpful and appreciated. Women had valuable interactions with kind and compassionate HCPs. Women found the ongoing contact with the bereavement midwives hugely beneficial. Access to early scans was a relief, easing first‐trimester anxiety. Continuity of care mattered as women did not have to retell their story, and they built a relationship with the HCP. Many women chose private obstetric care for this reason.

Women with less positive experiences of HCPs ‘explored all avenues’ of support, including online forums, support groups and private counselling. The main appeal of online forums was the opportunity to meet people with similar experiences who understood the journey, could offer advice on investigations and treatments, and reassure them of positive outcomes. Support groups offered similar opportunities but sometimes were not held locally, or included women without the dual experience or with living children. Podcasts and online support groups were also helpful. All supports helped to mitigate feelings of loneliness and isolation.

##### 3.1.4.3 Sub‐Theme 4.3 Staff Wanted to Do More for Women

Staff working with women with RM and infertility understood the complexities of their journey, especially the limitations of services and the pitfalls of care between the public and private sectors. Across the themes, HCPs spoke of ‘minding’ women with RM and infertility; this spoke to the vulnerability of this cohort in trying to have a baby, the lack of regulation within the private sector, and the expense involved. They endeavoured to support women with education, reducing their administrative burden and maximising what investigations and support they could offer publicly. HCPs in the private sector worried about women needing emergency miscarriage care in public hospitals out of hours and that women may not meet HCPs who understood the complexity of their situation. HCPs caring for women in subsequent pregnancy recognised that the anxiety persisted beyond the first trimester and offered frequent visits, early induction or Caesarean delivery.

HCPs' future care aspirations were dependent on services currently available. For HCPs in peripheral units, improvements would commence with recruiting a consultant specialised in fertility or RM to provide standardised care. In units with established practices, HCPs wished to streamline referral pathways and expand existing services to accommodate women. Most HCPs welcomed the new fertility hubs, but recognised the limitations of the strict criteria for ART and that women in western Ireland would remain geographically disadvantaged. They highlighted the difficulties in recruiting specialised staff in embryology and andrology to public services as potentially problematic, alongside nursing staff. HCPs felt strongly that a public service should not be a substandard service.

## 4 Discussion

This qualitative study examined the lived experience of RM and infertility through 33 semi‐structured interviews with women and HCPs across the ROI. Through Reflexive Thematic Analysis, we generated four interrelated themes that illuminate the complexity of this experience for women/couples, which impacted all areas of their lives. This was compounded by the challenges presented by deficits and divisions within the healthcare system and society. The experience separated them from other people and, within the system, perpetuating loneliness and feelings of isolation.

### 4.1 Strengths and Limitations

This study is novel in exploring the views and experiences of women with RM and infertility and HCPs. This is a large study of women and HCPs with various lived and clinical experiences, across a range of geographical locations, and inclusive of public and private health sectors, which substantially contributes to the literature. While the Irish health service issues are unique, the findings are transferable as ART is increasingly self‐funded internationally, and the experiences of RM and infertility are universal. A limitation is that the women interviewed were all white Irish, had high educational attainment in professional employment, and had access to private services, which may not reflect experiences in other socio‐economic groups. While the geographical spread of participants was not more equal, it does reflect the population spread in the ROI. While HCPs had current and past experiences within private fertility services, there was poor engagement from private services, limiting insights into care structures. This was despite email reminders and efforts associated with higher response rates, for example, personalisation [39].

### 4.2 Comparison to Wider Literature

This study aligns with other qualitative studies examining the dual experience in demonstrating a profound impact on the lives of women and their partners—psychologically, socially and financially [8, 12, 13, 14]. While women with RM and infertility can identify with either individual experience, the combined experience has distinct adversities [12]. The grief of loss is augmented by requiring ART and contemplating restarting treatment to become pregnant again [12, 13]. Perseverance requires that hopelessness of repeated losses and failed treatments is buoyed by the hope for a successful pregnancy, an internal struggle described previously [12, 40]. These specific hurdles, and the valid fear of never having children, contributed significantly to the psychological impact [12]. Women in our study also experienced anxiety and depression [8, 11, 14]. We too identified that feeling separate from others and the stigma associated with RM and infertility augmented women's loneliness, which further affected mental health [9, 12, 13]. A lack of understanding from other people and unhelpful comments [12, 13, 41], including from HCPs [8, 41, 42], were common findings.

Stressors previously identified, such as geographical disparities, cost and reproductive health leave, were also evident [8, 12, 41]. While logistical issues related to a public/private divide were specific to the ROI, Berger et al. [41], in their qualitative study of US women undergoing ART, identified a similar stress when women were ‘caught in a web’ of multiple medical providers. Our work uniquely explores HCPs' experiences of managing RM and infertility. HCPs across both sectors were sensitive to the burden of the dual experience and felt obligated to counsel women comprehensively, hoping to ‘protect’ women against unnecessary expense or malign practices. Again, HCP factors such as awareness and empathy influenced women's care experiences [8, 12, 41, 42].

This study distinctly identified that women with RM actively seek fertility care to improve their pregnancy chances. This was alluded to by Ozturk et al., but unexplored. Berger et al. [41] identified the coping phenomenon of ‘taking action’, such as researching treatments, to overcome anxiety related to infertility, which is evident in ‘exploring all avenues’ and ‘exhausting all resources’. Additional common coping strategies included seeking out others with similar experiences online for emotional support and reassurance [8, 12]. Women valued knowledge and competence in HCPs and continuity of care [36, 43]. Women described similar care needs to those within the literature, specifically, a need for time and sensitivity, compassion, and separate clinical spaces to pregnant women [12, 36, 40, 44, 45, 46]. These recurrences reflect the lack of priority and meaningful action afforded to women's health issues in policy and practice [47, 48].

Further issues considered by participants included unawareness of RM and infertility and related risk factors, deferred parenthood and societal influences/pressures [4, 49, 50, 51]. They identified that current government funding is insufficient for equitable access to ART. Participants proposed improving awareness of infertility through public health awareness campaigns and greater education in schools, including success rates of ART and oocyte preservation, as in Scandinavia [50, 51]. Another deficiency identified was protected reproductive health leave to mitigate work‐related pressures for women [52, 53].

### 4.3 Questions Which Remain to be Answered

While this study provides insights into the male experience from the perspectives of women and HCPs, there is a need for qualitative studies on the impact of infertility and RM on men and how supports are best delivered. For women/couples who do not subsequently have a child following RM and infertility, better support is required to navigate the impacts identified, such as grief and trauma. Further qualitative work should explore their distinct care needs.

The number of women/couples affected in the ROI remains unclear, due to the lack of a public register/database recording the numbers of people experiencing miscarriage or ART and related outcomes. Such information would allow for better advocacy for resources for maternity services and required supports, alongside better insights into treatment efficacy. While new National Clinical Guidelines have been published for RM and fertility [18, 54, 55], further high‐quality evidence is needed to advance management.

### 4.4 Conclusion

In summary, this study provides rich insights into the experiences of RM and infertility. As identified by women and HCPs, this complex experience is intensified by the division of care within the healthcare system and a lack of awareness of these reproductive health issues. The profound grief and emotional impact were compounded by a lack of understanding and support. Greater education, improved information provision and better support, together with enhanced networking, communication and care pathways between the public and private sectors, are needed.

## Author Contributions

**Laura Aoife Linehan:** writing – original draft, writing – review and editing, formal analysis, investigation, project administration. **Marita Hennessy:** writing – review and editing, methodology, conceptualisation, investigation, formal analysis. **Keelin O'Donoghue:** conceptualisation, writing – review and editing, methodology.

## Ethics Statement

Ethical approval for the study was granted by the Clinical Research Ethics Committee of the Cork Teaching Hospitals [Reference number: ECM 4 (J) 28/06/2022 and ECM 3 (eee) 13/12/2022].

## Consent

All participants gave informed written consent before the interview.

## Conflicts of Interest

The authors declare no conflicts of interest.

## Supporting information

Supplementary File 1.

Supplementary File 2.

Supplementary file 3.

## Data Availability

The data that support the findings of this study are available on request from the corresponding author. The data are not publicly available due to privacy or ethical restrictions.
