# Supplementary material for: Recurrent Miscarriage and Infertility Services and Supports: A Qualitative Study of Views and Experiences in the Republic of Ireland
Source: Health Expect. 2025 Aug 19;28(4):e70396. doi: 10.1111/hex.70396 (PMC12362763; doi:10.1111/hex.70396)
Supplement: Supplementary file 1 [file HEX-28-e70396-s002.docx]

| Health professionals | Women with lived experience of recurrent miscarriage and infertility |
| --- | --- |
| Roles and responsibilities  Structure of care  Management of RM and infertility  Knowledge and understanding  Impact and support  Recommendations | Expectations of pregnancy before infertility and/or loss  Experience and management of infertility  Experience and management of recurrent miscarriage  Subsequent pregnancy or current fertility intentions  Knowledge and understanding  Impact and support  Recommendations |

**Supplementary File 1. Topic guide structure**
