# Supplementary material for: Recurrent Miscarriage and Infertility Services and Supports: A Qualitative Study of Views and Experiences in the Republic of Ireland
Source: Health Expect. 2025 Aug 19;28(4):e70396. doi: 10.1111/hex.70396 (PMC12362763; doi:10.1111/hex.70396)
Supplement: Supplementary file 2 [file HEX-28-e70396-s001.docx]

**Supplementary File 2. Setting**

Every woman who is pregnant and ordinarily resident in Ireland is entitled to maternity care under the Maternity and Infant Scheme^1^. Care is provided in 19 maternity units; four larger units (>6,000 births per annum), six medium (2000-6000 births) and nine small (<2000 births)^2^. The medium and small units can transfer high-risk or critically ill women to the larger units for neonatal or obstetric care. All hospitals currently provide public and private maternity care^3^.

Just 9/19 units have a dedicated RM clinic, with some units also without a clinical lead in pregnancy loss or a Clinical Midwife Specialist in Bereavement and Loss^4,5^. Prior to the publication of the National Clinical Guideline for RM in January 2023, the main clinical guideline in use by practitioners was the Royal College of Obstetrics and Gynaecology Guideline which defined RM as three consecutive miscarriages^6^. Five maternity units have a public fertility service which provides basic investigations and treatments^7^. A National Clinical Practice Guideline for Investigation and Management of Fertility in Secondary Care was published in October 2023^7^, and the Clinical Practice Guideline for Artificial Reproductive Technology (ART) is expected in late 2024. Up to September 2023, the ROI was an outlier in Europe for not having publicly funded in-vitro fertilisation (IVF) with reimbursement limited to tax-relief on medical expenses and medications required^8,9^. While publicly funded IVF commenced in September 2023, much has yet to be established and is limited to three cycles of intra-uterine insemination (IUI) in addition to one round of IVF where women are aged under 40, with a BMI of <30 with no living children and no prior IVF treatment^10^. While a sixth maternity unit will also provide these services, geographical disparity of access to fertility care remains. In view of the above restrictions, most ART continues to be provided within private or not-for-profit fertility centres, of which there are currently nine main providers. These are largely located in urban areas, predominantly in the east of the country.

1. Maternity Care - HSE.ie. Accessed October 27, 2023. https://www.hse.ie/eng/services/list/3/maternity/

2. National Perinatal Epidemiology Centre. SEVERE MATERNAL MORBIDITY in Ireland 2021. Published online 2021. Accessed October 27, 2023. www.ucc.ie/en/npec/

3. Reynolds CME, McMahon LE, O’Malley EG, O’Connell MP, Sheehan SR, Turner MJ. Trends in private maternity care in Ireland’s capital during and after the Great Economic Recession 2009–2017. *Ir J Med Sci*. 2021;190(3):933. doi:10.1007/S11845-020-02415-Z

4. Hennessy M, Linehan L, Flannery C, Cotter R, O’connell O, O’donoghue K. A National Evaluation of Recurrent Miscarriage Care Services. *Ir Med J*. 2023;116(1):2023.

5. ON THE IMPLEMENTATION OF THE NATIONAL STANDARDS FOR BEREAVEMENT CARE FOLLOWING PREGNANCY LOSS AND PERINATAL DEATH. Published online 2021.

6. Royal College of Obstetricians and Gynaecologists. *Green-Top Guideline Number 17. The Investigation and Treatment of Couples with Recurrent First-Trimester and Second-Trimester Miscarriage*.; 2011.

7. National Clinical Practice Guideline – Fertility – Investigation and Management in Secondary Care. Accessed December 12, 2023. https://www.rcpi.ie/faculties/obstetricians-and-gynaecologists/national-clinical-guidelines-in-

8. Timoney A. *Data: Assisted Human Reproduction*.; 2022.

9. ESHRE. *A Policy Audit on Fertility*.; 2017. Accessed October 1, 2020. http://www.fertilityeurope.eu/our-projects/policy-audit/

10. Getting IVF and other specialist treatment through the HSE - HSE.ie. Accessed October 31, 2023. https://www2.hse.ie/pregnancy-birth/trying-for-a-baby/your-fertility/getting-ivf-icsi-iui-hse/
