# Supplementary material for: Recurrent Miscarriage and Infertility Services and Supports: A Qualitative Study of Views and Experiences in the Republic of Ireland
Source: Health Expect. 2025 Aug 19;28(4):e70396. doi: 10.1111/hex.70396 (PMC12362763; doi:10.1111/hex.70396)
Supplement: Supplementary file 3 [file HEX-28-e70396-s003.docx]

| Phase | Description |
| --- | --- |
| Familiarisation | Transcripts were verified against the recording by LL and anonymised. All transcripts were read by LL and some by MH. |
| Coding the data | Inductive open coding was initiated, which facilitated the early identification of meaning and ideas. These were then revised, re-named and re-grouped after reflection and discussion. |
| Generating initial key themes | Codes with participants’ common ideas or meanings were initially clustered into categories and tentative themes. These patterns were discussed and evaluated in thematic maps. |
| Re-evaluation of the themes | Categories and themes were reconsidered in visual mapping. Codes and raw data were revisited to ensure that deeper meanings were extracted and that meanings were retained in generating themes. |
| Defining and naming themes | Themes were refined to ensure that each theme was distinct with no overlap, but that each theme was related and integrated the overall experience. |
| Writing the report | The article was written up to form part of a Doctoral thesis and also presented locally, nationally and internationally to healthcare professionals working in the field. |

**Supplementary file 3. Reflexive thematic analysis**
